# Background review for diagnostic test development for Zika virus infection

**DOI:** 10.2471/BLT.16.171207

**Published:** 2016-08-01

**Authors:** Rémi N Charrel, Isabelle Leparc-Goffart, Suzan Pas, Xavier de Lamballerie, Marion Koopmans, Chantal Reusken

**Affiliations:** a UMR EPV Emergence des Pathologies Virales, Aix Marseille Université, Marseille, France.; b French National Reference Centre for Arbovirus, Armed Forces Biomedical Research Institute, Marseille, France.; c Department of Viroscience, WHO Collaborating Centre for Arboviruses and Viral Haemorrhagic Fever Research and Reference, Wytemaweg 80, Room EE 17-26, 3015 CN Erasmus MC, Rotterdam, Netherlands.; Correspondence to Chantal Reusken (email: c.reusken@erasmusmc.nl).

## Abstract

**Objective:**

To review the state of knowledge about diagnostic testing for Zika virus infection and identify areas of research needed to address the current gaps in knowledge.

**Methods:**

We made a non-systematic review of the published literature about Zika virus and supplemented this with information from commercial diagnostic test kits and personal communications with researchers in European preparedness networks. The review covered current knowledge about the geographical spread, pathogen characteristics, life cycle and infection kinetics of the virus. The available molecular and serological tests and biosafety issues are described and discussed in the context of the current outbreak strain.

**Findings:**

We identified the following areas of research to address current knowledge gaps: (i) an urgent assessment of the laboratory capacity and capability of countries to detect Zika virus; (ii) rapid and extensive field validation of the available molecular and serological tests in areas with and without Zika virus transmission, with a focus on pregnant women; (iii) monitoring the genomic diversity of circulating Zika virus strains; (iv) prospective studies into the virus infection kinetics, focusing on diagnostic sampling (specimen types, combinations and timings); and (v) developing external quality assessments for molecular and serological testing, including differential diagnosis for similar viruses and symptom clusters. The availability of reagents for diagnostic development (virus strains and antigens, quantified viral ribonucleic acid) needs to be facilitated.

**Conclusion:**

An international laboratory response is needed, including preparation of protocols for prospective studies to address the most pressing information needs.

## Résumé

**Objectif:**

**Faire le point sur l'état des connaissances concernant les tests de diagnostic de la maladie à virus Zika et déterminer les domaines de recherche à approfondir pour combler les lacunes actuelles en la matière.**

**Méthodes:**

**Nous avons procédé à une revue non systématique de la documentation publiée au sujet du virus Zika, que nous avons complétée par des renseignements issus de kits de diagnostic du commerce et de communications personnelles avec des chercheurs travaillant dans des réseaux européens de préparation aux épidémies. Lors de cette revue, nous avons examiné les connaissances actuelles sur la couverture géographique, les caractéristiques pathogènes et le cycle de vie du virus, ainsi que sur la cinétique d'infection par celui-ci. Les tests moléculaires et sérologiques disponibles, tout comme les questions de biosécurité, sont décrits et abordés dans le cadre de la flambée actuelle et de sa souche.**

**Résultats:**

**Nous avons identifié les domaines de recherche suivants à approfondir pour combler les lacunes qui existent à l'heure actuelle: (i) évaluation urgente des moyens des laboratoires et des capacités des pays à détecter le virus Zika; (ii) validation rapide et généralisée sur le terrain des tests moléculaires et sérologiques disponibles dans les zones touchées ou non par la transmission du virus Zika, notamment pour les femmes enceintes; (iii) surveillance de la diversité génomique des souches du virus Zika en circulation; (iv) études prospectives sur la cinétique de l'infection par le virus, axées sur l'échantillonnage de diagnostic (types d'échantillons, combinaisons et chronologie); (v) élaboration d'évaluations externes de la qualité des tests moléculaires et sérologiques, avec diagnostic différentiel par rapport aux virus similaires et aux symptômes. Il est nécessaire de faciliter l'accès à des réactifs pour la réalisation du diagnostic (souches du virus et antigènes, quantification de l'acide ribonucléique viral).**

**Conclusion:**

**Une intervention des laboratoires est nécessaire à l'échelle internationale, notamment pour préparer des protocoles d'études prospectives permettant de répondre aux besoins les plus urgents en matière d'information.**

## Resumen

**Objetivo:**

Revisar el estado del conocimiento sobre las pruebas de diagnóstico para la infección por el virus de Zika e identificar las áreas de investigación necesarias para abordar las actuales lagunas en los conocimientos.

**Métodos:**

Se llevó a cabo una revisión no sistemática de las publicaciones sobre el virus de Zika y se complementó con información derivada de equipos de pruebas de diagnóstico comerciales y comunicaciones personales con investigadores de redes de preparación europeas. La revisión abarcó el conocimiento actual sobre la propagación geográfica, las características patógenas, el ciclo de vida y la cinética de infección del virus. Las pruebas moleculares y serológicas disponibles, así como temas relativos a la bioseguridad, se describen y debaten en el contexto de la cepa del brote actual.

**Resultados:**

Se identificaron las siguientes áreas de investigación para abordar las actuales lagunas en los conocimientos: (i) una evaluación urgente de la capacidad de laboratorio y de los países para detectar el virus de Zika; (ii) una validación de campo rápida y extensa de las pruebas moleculares y serológicas disponibles en zonas con y sin la transmisión del virus de Zika, prestando especial atención a las mujeres embarazadas; (iii) un seguimiento de la diversidad genómica de las cepas del virus de Zika activas; (iv) estudios prospectivos sobre la cinética de infección del virus, con especial atención a las muestras de diagnóstico (tipos de espécimen, combinaciones y tiempos); y (v) desarrollo de evaluaciones de calidad externas para las pruebas moleculares y serológicas, incluidos diagnósticos diferenciales para virus similares y conjuntos sintomáticos. Es necesario facilitar la disponibilidad de reactivos para desarrollar los diagnósticos (cepas del virus y antígenos, ácido ribonucleico viral cuantificado).

**Conclusión:**

Se necesita una respuesta de laboratorios internacionales, incluida la preparación de protocolos para estudios prospectivos, a fin de satisfacer las necesidades informativas más urgentes.

## ملخص

**الغرض:**

مراجعة الحالة المعرفية بالاختبارات المخصصة لتشخيص العدوى بفيروس زيكا وتحديد مجالات البحوث اللازمة للتعامل مع القصور الحالي في المستوى المعرفي.

**الطريقة:**

قمنا بإجراء مراجعة بصورة غير منهجية للكتابات المنشورة حول فيروس زيكا وألحقنا ذلك بمعلومات من أدوات الاختبار التشخيصي التجارية والمقابلات الشخصية مع الباحثين في الشبكات الأوروبية التي هي على قدر من التأهب. وقد تناولت المراجعة مستوى المعرفة الحالي بمدى الانتشار الجغرافي للفيروس، وخصائص العوامل المسببة للأمراض فيه، ودورة حياته، وسماته الحركية المسببة للعدوى. وتم تحليل الاختبارات المتاحة المتعلقة بالجزيئات واستخدام الأمصال بجانب مشكلات السلامة البيولوجية ومناقشتها في ضوء السلالة الحالية المتفشية من الفيروس.

**النتائج:**

قمنا بتحديد المجالات البحثية التالية اللازمة للتعامل مع القصور الحالي في المستوى المعرفي: ضرورة التقييم العاجل لمستوى الإمكانيات بالمختبرات وقدرة البلدان على اكتشاف عدوى فيروس زيكا؛ و(2) التحقق الميداني السريع والشامل من صحة الاختبارات المتاحة المتعلقة بالجزيئات واستخدام الأمصال التي يتم إجراؤها في مناطق مؤهلة وغير مؤهلة لانتشار فيروس زيكا فيها مع صب التركيز على السيدات الحوامل؛ و(3) مراقبة التنوع الجيني في سلالات فيروس زيكا المنتشرة؛ و(4) إجراء دراسات استباقية حول سمات الفيروس الحركية المسببة للعدوى مع التركيز على استخدام العينات التشخيصية (أنواع العينات والمجموعات والتوقيتات)؛ و(5) تطوير التقييمات الخارجية لجودة الاختبارات المتعلقة بالجزيئات واستخدام الأمصال بما في ذلك التشخيصات المتباينة لفيروسات مشابهة ومجموعات من الأعراض. الكاشفات المسؤولة عن التطور التشخيصي (سلالات الفيروس ومُستضداته، والحمض الرّيبي النووي للفيروس الذي يتم تحديد مقداره) بحاجة إلى تيسير مدى توافرها.

**الاستنتاج:**

يلزم وجود استجابة دولية بخصوص المختبرات بما في ذلك التحضير للبروتوكولات اللازمة للدراسات الاستباقية لتلبية أكثر الرغبات الملحّة للحصول على معلومات. ****

## 摘要

**目的:**

旨在对寨卡病毒感染诊断试验的知识状态进行综述，并确定要弥补当下知识缺口所需的研究领域。

**方法:**

我们对已出版的关于寨卡病毒的文献进行了非系统化综述，并且利用商业诊断试剂盒上的信息以及同欧洲技术解答网络研究人员的个人交流信息对其进行了补充。 本综述内容包括该病毒的地理分布、病原体特性、生命周期以及传染动力学相关知识。 文章描述并讨论了在当前爆发菌株的背景下可实现的分子学以及血清学试验。

**结果:**

我们确定了以下研究领域以弥补当前的知识空缺：(i) 对各国实验室检测寨卡病毒的能力进行紧急评估；(ii) 在有和没有寨卡病毒传播的区域对可开展的分子学和血清学试验进行快速、广泛的实地验证，重点关注孕妇群体。(iii) 监控传播中的寨卡病毒菌株的基因组多样性；(iv) 开展病毒传染动力学前瞻研究，重点关注诊断用取样（样本类型、样本组合以及取样时间）；以及 (v) 对分子学和血清学试验（包括相似病毒以及症状群集的鉴别诊断）进行外部质量评估。 亟待提升诊断研发（病毒菌株和病原体、量化的病毒核糖核酸）试剂的可获取性。

**结论:**

需要建立一套国际性实验室响应机制，包括制定解决最紧急信息需求所需的前瞻研究方案。

## Резюме

**Цель:**

Проанализировать изученность тестирования для диагностики заражения вирусом Зика и определить, в какие области исследования необходимо направить деятельность, чтобы восполнить имеющиеся пробелы в знаниях.

**Методы:**

Авторы провели несистематический обзор опубликованной литературы, посвященной вирусу Зика, и объединили эту информацию с информацией, полученной с помощью коммерческих диагностических тест-наборов и в ходе личного общения с исследователями в европейских системах готовности. В обзор были включены существующие знания о географическом распространении, особенностях возбудителя, жизненном цикле и кинетике вируса. Доступные молекулярные и серологические методы тестирования, а также проблемы в области биобезопасности описаны и изучены в контексте циркулирующего сегодня эпидемического штамма.

**Результаты:**

Было определено, что в настоящий момент пробелы в знаниях имеются в следующих областях исследования: (i) неотложная оценка лабораторного потенциала и способности стран выявлять вирус Зика; (ii) быстрое и исчерпывающее подтверждение результатов доступных молекулярных и серологических тестов в полевых условиях как в регионах распространения вируса Зика, так и во всех остальных с выделением беременных женщин в группу особого внимания; (iii) отслеживание геномного разнообразия циркулирующих штаммов вируса Зика; (iv) проспективные исследования кинетики вируса, акцентирующие внимание на взятии проб для диагностики (виды, сочетания проб и сроки их взятия); (v) разработка процедур внешней оценки качества молекулярных и серологических методов тестирования, в том числе дифференциальной диагностики для схожих вирусов и симптомокомплексов. Необходимо увеличить доступность реагентов для развития диагностики (штаммы и антигены вируса, вирусная рибонуклеиновая кислота в количественном выражении).

**Вывод:**

От лабораторий всего мира требуются ответные действия, включающие подготовку инструкций для проспективных исследований, которые позволили бы удовлетворить наиболее неотложные потребности в информации.

## Introduction

On 1 February 2016, the World Health Organization (WHO) declared that the recent cluster of microcephaly cases and other neurological disorders reported in the Americas, where an outbreak of Zika virus infection is ongoing, constitutes a public health emergency of international concern.^1^

Zika virus is a mosquito-borne virus related to yellow fever, dengue, West Nile, Japanese encephalitis and tick-borne encephalitis viruses which all belong to the virus family *Flaviviridae* and genus *Flavivirus*. Flaviviruses are positive-sense ribonucleic acid (RNA) viruses, with a genome of approximately 11 kilobases. Virions are produced as spherical particles, 40–60 nm in diameter.^2^ The virus was first isolated in 1947 from rhesus monkeys living in the Zika forest in Uganda.^3^ Up to 2006, only sporadic cases of humans infected with the virus had been reported in the literature.^4^ Accordingly, Zika virus was long considered a low-impact human pathogen, until outbreaks were reported in Yap Island in the Federated States of Micronesia in 2007 with 118 confirmed and suspected cases^5^ and in French Polynesia in 2013–2014 with an estimated 30 000 cases.^6^ This might explain the limited number of articles listed in the PubMed database up to 21 January 2016 (269) compared with that for other mosquito-borne viruses such as dengue virus (9187), West Nile virus (5949) and chikungunya virus (2183).^3^^,^^4^

Zika virus infection is often asymptomatic; around 80% of patients showed few or no clinical symptoms during the outbreaks in Yap Island and French Polynesia.^5^^,^^6^ The incubation period for the infection was estimated to range from 3 to 12 days.^6^^–^^8^ Common symptoms in confirmed patients are a maculopapular rash (90–96% of cases), fever (62–65%), myalgia and arthralgia (48–65%), headache (45–58%), non-purulent conjunctivitis (38–55%) and retro-orbital pain (40%).^6^^–^^8^ Interim case definitions were published by WHO on 12 February 2016.^9^

A major concern is the possible association of Zika virus infection with Guillain–Barré syndrome – a neurological disorder – and with microcephaly and other neurological manifestations in newborns of infected mothers. These complications have been noticed in the current epidemic region and were identified retrospectively in the French Polynesia outbreak.^8^^,^^10^ However, the question arises whether the increased incidences of Guillain–Barré syndrome and microcephaly in the current outbreak are due to certain specific virulent strains or to a common pattern of all Zika virus strains that have gone unnoticed because of the low number of cases in previous outbreaks.^8^ In Brazil, more than 4700 cases of suspected microcephaly were recorded from mid-2015 to the end of January 2016, whereas the number usually is below 200 cases per year.^11^ Brazil, the Bolivarian Republic of Venezuela, Colombia, El Salvador and Suriname reported spikes in Guillain–Barré syndrome cases in January 2016.^12^^,^^13^ While it remains to be determined if Zika virus infection causes these complications, several governments and health agencies have issued precautionary travel advice for the affected regions, with specific information for pregnant women.^14^

The Zika virus outbreak is also likely to increase the number of cases exported from epidemic areas by travellers (Table 1). The current epidemic has therefore resulted in a large increase in requests for laboratory diagnosis of suspected cases of Zika virus infection not only among residents of the outbreak region but also among travellers returning from affected areas, especially pregnant women with or without current or past clinical symptoms of a Zika virus infection. We therefore identified a need to assess the current state of preparedness in laboratory diagnostics to ensure a timely and accurate response to the Zika virus outbreak in both affected and unaffected regions. 

**Table 1:** Molecular and serological diagnosis of cases of Zika virus imported by patients travelling from outbreak areas, 2013–2015

Country imported into	Country or island imported from	No. of human cases	Zika virus RNA detection results	Positive Zika virus serology results
Australia^15^	Cook Islands	1	Serum-positive	IgG, IgM seroconversion
Australia^16^	Indonesia	1	Serum-positive	–
Canada^17^	Thailand	1	Serum-, urine-positive	IgM; seroconversion in neutralization assay
Finland^18^	Maldives	1	Urine-positive	Not tested
Germany^19^	Thailand	1	Serum-negative	IgM, IgG
Germany^20^	Indonesia	1	Serum-negative	IgG, IgM seroconversion; neutralization assay
Italy^21^	Brazil	1	–	IgM; IgG seroconversion; seroconversion neutralization assay
Italy^22^	French Polynesia	2	Serum-positive	IgG, IgM seroconversion
Japan^23^	Thailand	1	Serum-equivocal,^a^ urine-positive	IgM
Japan^24^	French Polynesia	2	Serum-, urine-positive	IgM, seroconversion neutralization assay
Norway^25^	French Polynesia	1	Serum-positive	IgG, IgM seroconversion
United States^26^	French Polynesia	1	–	IgM; IgG seroconversion

Ig: immunoglobulin: RNA: ribonucleic acid.

^a^ Discrepant results observed between two assays.

Note: Dashes indicate data not described.

## Methods

We made a non-systematic review to present the essential background information and current gaps in knowledge about diagnostic testing for Zika virus infection in humans. We made a literature search of the PubMed database up to 21 January 2016, using the search terms “Zika virus”, “ZIKV” or “Zika”, with no date or language restriction. This was supplemented by information obtained from commercially available diagnostic test kits and from personal communications with researchers in European preparedness networks. We identified areas of research and actions needed to address the identified gaps in international laboratory preparedness for an adequate response to the current outbreak.

## Epidemiology

### Geographical spread

Serological, epidemiological and entomological studies have reported the circulation of the virus in tropical areas of western Africa, central Africa and in Asia.^3^ By 14 April 2016, Zika virus autochthonous transmission had been reported in 35 countries in the Americas.^27^ The increased risk of importing Zika virus to Europe is illustrated by recent reports of cases of the virus in travellers to many European countries.^28^

### Pathogen and transmission pathways

**Fig. 1:**
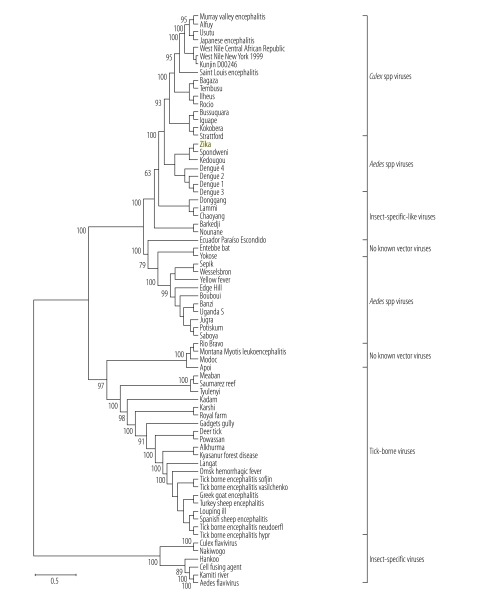
Phylogenetic relationships among representative viruses within the family *Flaviviridae* based on complete genomic sequence analysis

Notes: Sequences were aligned using the Clustal W program (EMBL-EBI, Cambridgeshire, United Kingdom). Distances and groupings were determined by the Kimura-2 parameter method and the neighbour-joining algorithm implemented with the pairwise deletion model in the Molecular Evolutionary Genetics Analysis (MEGA) version 6.06 software program. Percentage bootstrap values are indicated at the branch nodes. The scale at the bottom of the tree indicates the number of nucleotide substitutions per site.

**Fig. 2:**
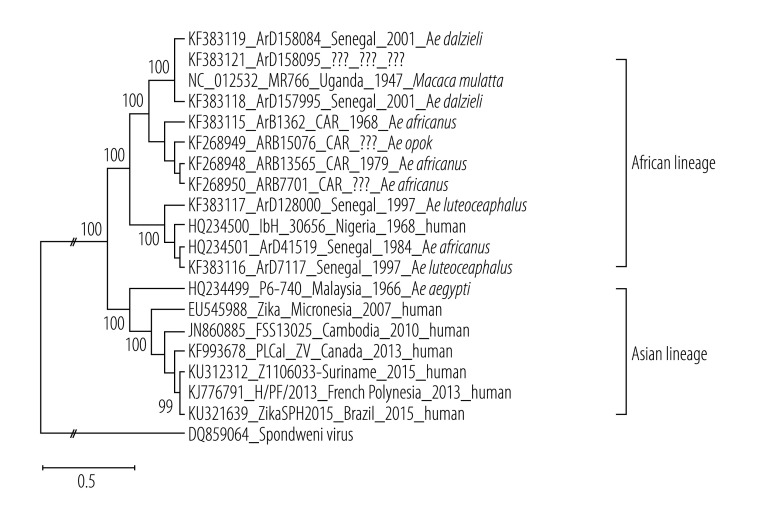
Phylogenetic relationships among selected Zika virus strains belonging to the African and Asian lineages based on complete genomic sequence maximum (likelihood analysis)

Notes: The tree was performed with 19 complete sequences available in the GenBank® database (National Institutes of Health, Bethesda, USA) as of 21 January 2016, together with a sequence of the closely related Spondweni virus as outgroup. Sequences were aligned using the Clustal W program. The tree was built using the maximum likelihood method with the best fitted parameters calculated in the MEGA version 6.06 software program (general time reversible model with gamma distribution with invariant sites and nearest-neighbour-interchange using a very strong branch swap filter). The sequences are labelled with the following information: GenBank® accession number_strain_country_year of isolation_host. Percentage bootstrap values are indicated at the branch nodes. The scale at the bottom of the tree indicates the number of nucleotide substitution per site.

Zika virus belongs to the Spondweni virus serogroup of mosquito-borne flaviviruses (Fig. 1). Phylogenetic analysis reveals the existence of two lineages (Fig. 2): the African lineage, which has shown no propensity to disseminate outside of Africa, and the Asian lineage, which continues to seed in previously unaffected regions of the world.^3^ All recently disseminated strains belong to the Asian lineage.^29^^,^^30^ Zika virus genomes from patients infected in Brazil and Suriname in 2015 are closely related to the strain that circulated in French Polynesia in 2013 (Fig. 2), with more than 99.7% and 99.9% level of nucleotide and amino acid identities, respectively.^30^

Zika virus is transmitted by *Aedes* mosquitoes; *Ae. aegypti* is the only species for which transmission outside Africa has been confirmed. In the 2007 outbreak on Yap Island, *Ae. hensilii* mosquitoes were implicated as the vector, but this could never be confirmed by virus detection. The virus has been isolated and/or detected by reversed transcriptase (RT) polymerase chain reaction (PCR) assay from the following species in the field in Africa: *Ae. africanus*, *Ae. aegypti*, *Ae. albopictus*, *Ae. apicoargenteus*, *Ae. luteocephalus*, *Ae. vitattus, Ae. taylori*, *Ae. dalzieli*, *Ae. hirsutus, Ae. metallicus, Ae. unilinaetus, Ae. opok* and *Ae. furcifer*.^31^^,^^32^ In addition, genomic RNA was detected in Senegal in mosquitoes of three species: *Mansonia uniformis, Culex perfuscus* and *Anopheles coustani*. *Ae. albopictus* has shown competence in Zika virus dissemination in laboratory studies but has never been implicated in Zika virus epidemiology in the field outside of Africa.^32^^,^^33^

Additional modes of transmission have been identified. Perinatal transmission most probably occurs by transplacental transmission or during delivery by an infected mother.^34^ Sexual transmission has been indicated in multiple cases.^35^ Zika virus has been isolated from semen collected 14 days after the start of symptoms,^36^ while detection of the Zika virus genome was described in semen at 28 and 62 days after the onset of symptoms.^37^ The potential for Zika virus transmission via blood transfusion was identified in the French Polynesia outbreak in 2013–2014^38^ and the Brazilian authorities announced the first confirmed cases of blood-transfusion mediated transmission on 5 February 2016.^39^

### Infection kinetics

Knowledge of the Zika virus infection kinetics is essential to determine the optimal strategy for diagnosis. This is hindered by the fact that only a few diagnosed cases of human Zika virus infection, all of Asian lineage, are described in the literature.

#### Presence of virus in specimens

Data from the French Polynesia outbreak described viraemia that was of low intensity and short duration.^40^^–^^42^ Zika virus has been detected in serum, saliva, urine and nasopharyngeal swabs by molecular methods. In serum, the virus can be detected typically up to 3–5 days after the onset of clinical symptoms; the viral load seems to peak when clinical signs appear.^41^^,^^43^ The time frame of detection in saliva is no longer than in serum. A study combining the diagnostic results from blood and saliva specimens from 182 suspected cases increased the rate of detection; 19% of the total of 103 positive cases were detected in saliva only while 52 cases were detected with both saliva and serum.^40^ Data from observations of six patients in the French territory of New Caledonia and from other case reports suggested that detection of Zika virus in urine should be supplemented by molecular testing of blood samples.^18^^,^^23^^,^^41^ The viral load in urine was higher than in blood, peaking at days 5–7, and seemed to last longer, with detection by PCR assay possible up to 20 days after clinical onset of Zika virus disease.^41^ In one case, Zika virus RNA was still detected in patient’s urine at day 28 after the onset of illness (authors’ own unpublished observations, Erasmus University Medical Centre, January 2016). Virus isolation from urine from a case using Vero cells has been described.^17^ Detection of Zika virus RNA in nasopharyngeal swabs when serum samples were negative was described in two cases.^17^^,^^44^ The low invasiveness of urine and saliva collection is an advantage for diagnosis of infants and newborns. More studies on detection of Zika virus in different types of specimens are needed, as these observations are based on a limited number of cases.

#### Immune response

Typically for flaviviruses, immunoglobulin (Ig) M antibodies develop within a few days after onset of illness and can generally be detected for up to 3 months. IgG antibodies develop within days after IgM and can be detected for months to years. Cases have been described with persistence of IgM antibodies for longer periods, which complicates accurate diagnostic testing.^45^

Immune response from Zika virus infection has only been described in 11 patients during the Zika virus outbreak on Yap Island. IgM was detected as soon as 3 days after the onset of symptoms. IgG appeared after day 10 in a patient with no history of previous flavivirus infections.^43^^,^^46^^,^^47^ In this patient, neutralizing antibodies against Zika virus could be detected as early as 5 days after the onset of fever.

Specific attention should be given in prospective studies to determine the Zika virus immune responses in pregnant women, since antibody responses during pregnancy may be different from those in non-pregnant women.^48^

## Laboratory diagnosis

To run molecular tests in an outbreak setting, laboratories need knowledge and experience of quality control and validation, access to rapid supply chains for reagents and plastics, and the capability to increase the throughput of testing. To speed up testing, experience with automated equipment for nucleic acid extraction and RT–PCR assay are essential.

In serological assays, extensive cross-reactivity between virus genus members or geographical overlap with other pathogens causing overlapping syndromes might occur. In these cases, pan-genus and syndromic serum panel tests and antigens need to be available and confirmatory techniques such as enzyme-linked immunosorbent assay (ELISA), immunofluorescence assay and virus neutralizing testing are required.

### Molecular methods

**Fig. 3:**
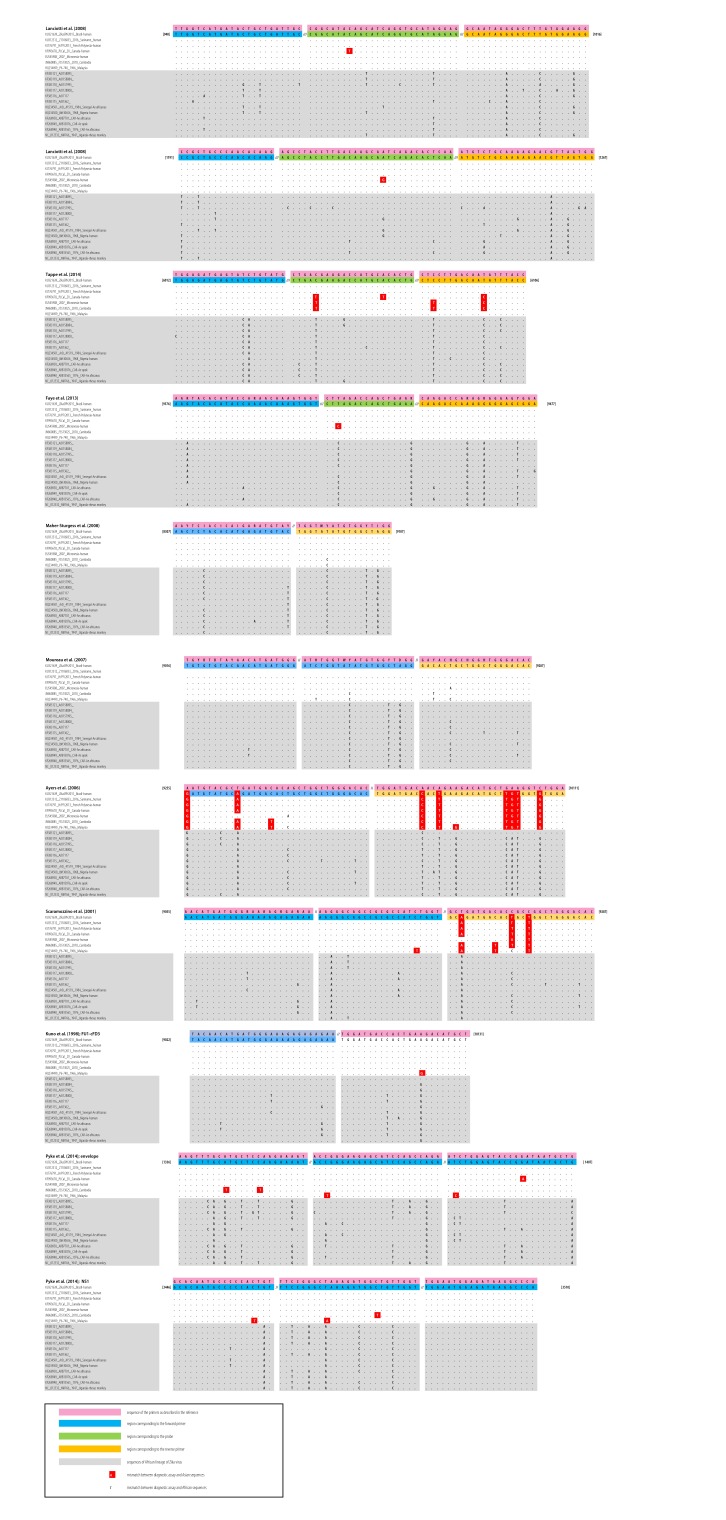
Reverse-transcription polymerase chain reaction methods and specific primers and probes used to detect cases of Zika virus infection in humans

Notes: The assays were performed with 19 complete sequences available in the GenBank® database as of 21 January 2016, together with a sequence of the closely related Spondweni virus as outgroup. Sequences were aligned using the Clustal W program. The numbers in brackets correspond to the nucleotide position in the reference sequence (ZikaSPH2015). The sequences are labelled with the following information: GenBank® accession number_strain_country_year of isolation_host.

Sources: Lanciotti et al. (2008);^43^ Tappe et al. (2014);^19^ Faye et al. (2013);^51^ Maher-Sturgess et al. (2008);^61^ Moureau et al. (2007);^56^ Ayers et al. (2006);^62^ Scaramozzino et al. (2001);^60^ Kuno et al. (1998);^58^ Pyke et al. (2014).^15^

Molecular diagnosis of Zika virus can be done on different types of body fluids: whole blood, serum, EDTA (ethylenediaminetetraacetic acid) plasma, saliva and urine. Urine and saliva should be considered together with blood and/or serum in the algorithm of Zika virus genome detection using molecular techniques. The reliance on the use of molecular diagnostics to rule out infection requires careful consideration, as the experience of clinicians and diagnostic laboratories is necessarily limited for emerging diseases. Several non-commercial RT–PCR tests for Zika virus have been described in the literature, but few provide validation using the most recent viral strains and fully documented clinical specimens. In this article, we only discuss the 12 RT–PCR assays that have resulted in the detection of viral RNA in at least one human case of Zika virus infection, as described in a peer-reviewed article indexed in PubMed or in a personal communication (Table 2). We have compared primers and probes used in these assays with the currently known Zika virus RNA sequences (Fig. 3; available at: http://www.who.int/bulletin/volumes/94/8/15-171207).

**Table 2:** Summary of the 12 reverse transcription polymerase chain reaction assays and sample types used to detect viral RNA in at least one human case of Zika virus infection

Author (year) of published PCR assay	PCR target	PCR technique	Amplicon size (bp)	Zika virus lineage analytical	Zika virus lineage field	No. of human patients tested in studies	Sample types positive in field
Lanciotti et al. (2008)^43^	Zika virus prM/E, target 1	Hydrolysis probe	76	Asian, African	Asian	> 200 (combined set)^5^^,^^10^^,^^23^^,^^24^^,^^34^^,^^36^^,^^38^^,^^40^^–^^42^^,^^49^^,^^50^	Serum, urine, amniotic fluid
Lanciotti et al. (2008)^43^	Zika virus E, target 2	Hydrolysis probe	76	Asian, African	Asian	> 200 (combined set)^5^^,^^10^^,^^23^^,^^24^^,^^34^^,^^36^^,^^38^^,^^40^^–^^42^^,^^49^^,^^50^	Serum, urine, amniotic fluid
Faye et al. (2013)^51^	Zika virus NS5	Locked nucleic acid probe	102	Asian, African	African	3 (B Rockx, personal communication, February 2016)	Serum
Tappe et al. (2014)^19^	Zika virus NS3	Hydrolysis probe	94	Asian	Asian	5^19^^–^^22^^,^^25^	Serum
Faye et al. (2008)^52^	Zika virus E	Conventional	364	African	Asian	> 15 (combined set)^53^^,^^54^	Serum
Pyke et al. (2014)^15^	Zika virus NS1	Hydrolysis probe	65	Asian	Asian	1^55^	Serum
Pyke et al. (2014)^15^	Zika virus E	Hydrolysis probe	71	Asian	Asian	1^55^	Serum
Moureau et al. (2007)^56^	Flavivirus NS5	SYBR®-green-based	269–272	African	Asian	2^18^^,^^57^	Serum, urine
Kuno et al. (1998)^58^	Flavivirus NS5	Conventional	1079	Asian, African	Asian	51^59^	Serum
Scaramozzino et al. (2001)^60^	Flavivirus NS5	Conventional (semi-nested)	220	African	Asian	1^55^ (L Barzon, personal communication, February 2016)	Serum, urine
Maher-Sturgess et al. (2008)^61^	Flavivirus NS5	Conventional	800	African	Asian	1^15^	Serum
Ayers et al. (2006)^62^	Flavivirus NS5	Conventional	863	–	Asian	1^17^	Serum, urine, nasopharynx

Bp: base pairs; E: envelope structural protein; NS1: non-structural protein 1; NS3: non-structural protein 3; NS5: non-structural protein 5; PCR: polymerase chain reaction; prM: precursor to membrane protein M; RNA: ribonucleic acid.

Note: Dashes indicate data not described.

#### RT–PCR

##### Zika-virus-specific

Various real-time and conventional RT–PCR assays specific for Zika virus have been described (Table 2).^19^^,^^43^^,^^51^^,^^52^ Lanciotti at al.^43^ described a combination of two real-time PCR assays and this approach is the most commonly used for direct diagnosis of Zika virus. Two gene targets were described and equivocal positive results were mentioned that could be related to false positives; of 157 samples tested, 10 were positive for only one target while 17 were positive for both.^43^ It was not mentioned whether this was randomly observed with both assays. However, discrepant results were observed between the two targets in the French and Dutch reference laboratories as well (authors’ own unpublished observations). Primer/probe set 1 in combination with TaqMan® Fast Virus 1-Master Mix (Life Technologies, Bleiswijk, Netherlands) was found to be less sensitive than set 2 (decrease of about 3 cycle thresholds) and showed worse amplification plots with Zika viruses of African lineage. For set 2, African and Asian lineages of the virus had comparable sensitivity and amplification plots. Sequencing of PCR products demonstrated that positivity with only set 1 was shown to be due to false positives rather than a lack of sensitivity of set 2. Recently, this assay was used to detect Zika virus RNA in the amniotic fluid of two pregnant women, from the state of Paraiba, Brazil, whose fetuses were diagnosed with fetal microcephaly.^63^

Other researchers have described an assay using a locked nucleic acid probe in a real-time RT–PCR assay for detection of Zika virus in human serum (Table 2),^52^ although the probe should be FAM-CTYAGACCAGCTGAAG-BBQ, with the locked nucleic acids indicated in bold type (M Weidmann, University of Stirling, personal communication, January 2016). Although no studies have yet been published, several laboratories are currently identifying Zika virus infection in humans using this assay (B Rockx, Dutch National Institute for Public Health and the environment, personal communication, February 2016). In general, locked nucleic acid and minor groove binder probes are not the best choice in PCR screening assays, since only one mutation can result in false-negative results due to failure to detect the amplified product. In cases such as Zika virus, where very few sequence data are available and the grade of genome conservation is not known, it is generally recommended to use TaqMan® probes in quantitative RT–PCR.

Commercial RT–PCR assays for Zika virus are rapidly becoming available. However, until now the tests are for research purposes only. The primers and probe sequences in commercial kits are not usually publicly available, which precludes *in silico* assessment of their fit with the current Zika virus. Commercial kits on the market include: RealStar® Zika virus RT–PCR kit 1.0 (Altona Diagnostics GmbH, Hamburg, Germany), Genesig® Zika virus Advanced kit (Primerdesign Ltd, Birmingham, United Kingdom of Great Britain and Northern Ireland), MyBioSource Zika real time RT–PCR kit (MyBioSource Inc., San Diego, United States of America), Genekam Zika virus PCR (Genekam Biotechnology AG, Duisburg, Germany) and FTD Zika virus RT–PCR kit (Fast-Track Diagnostics, Esch-sur-Alzette, Luxembourg). Manufacturers should be encouraged to put detailed information about their primers and probes into the public domain, so that the performance of these can be evaluated continuously in the context of our evolving knowledge about the genomic diversity of Zika virus during the current outbreak.

##### Pan-flavivirus combined with sequencing

One real-time^56^ and several conventional^58^^,^^60^^–^^62^ pan-flavivirus (detecting all viruses of genus *Flavivirus*) RT–PCR assays have been used in combination with sequencing to detect human Zika virus cases (Table 2).^15^^,^^17^^,^^18^^,^^57^^,^^59^^,^^64^

### Serological methods

The serology of flaviviruses is complex due to extensive cross-reactivity between antibodies triggered by different flavivirus infections or by vaccination, even for viruses belonging to different serogroups. In addition, an acute flavivirus infection might boost cross-reactive antibodies due to previous infection with or vaccination against another flavivirus.^45^ Table 3 summarizes by continent which flaviviruses, other than Zika virus, may cause cross-reactivity in serological tests, due to endemic circulation or vaccination. This shows that patients in the current Zika virus outbreak areas are likely to have a high background exposure to other flaviviruses, such as *dengue*virus,**yellow fever*virus* and West Nile virus, whereas European travellers returning from the same areas may not. As a consequence, a high proportion of the Zika virus infections in the outbreak region will be secondary flavivirus infections that will complicate the serology.

**Table 3:** Flaviviruses likely to show cross-reactivity with Zika virus under serological testing due to vaccination or endemic circulation in the population, by continent

Virus	Africa	Asia	Central America and the Caribbean	Europe (returning travellers)	Europe	North America	Oceania	South America
**Vaccine-related**								
Yellow fever virus	+	–	–	+	–	–	–	+
Japanese encephalitis virus	–	+	–	+	–	–	+	–
Tick-borne encephalitis virus	–	–	–	+	+	–	–	–
Dengue fever virus	–	–	+^a^	–	–	–	–	–
**Endemic circulation**								
Yellow fever virus	+	–	–	–	–	–	–	+^b^
Dengue fever virus	+	+^b^	+^b^	–	–	–	+	+^b^
West Nile virus	+	+^b^	+	–	+	+	+	+
Japanese encephalitis virus	–	+^b^	–	–	–	–	+	–
Tick-borne encephalitis virus	–	+	–	–	+	–	–	–
Usutu virus	+	–	–	–	+	–	–	–
Saint Louis encephalitis virus	–	–	+	–	–	+	–	+
Rocio virus	–	–	–	–	–	–	–	+
Ilheus virus	–	–	–	–	–	–	–	+
Murray valley encephalitis virus	–	–	–	–	–	–	+^b^	–
Kyasanur forest disease virus	–	+	–	–	–	–	–	–
Alkhurma haemorrhagic fever virus	–	+^c^	–	–	–	–	–	–
Wesselsbron virus	+	–	–	–	–	–	–	–

+: virus circulating on continent and could be cross-reactive; –: virus not circulating on continent.

^a^ Only in Mexico and possibly Brazil in near future (H Zeller, European Centre for Disease Prevention and Control, personal communication, February 2016).

^b^ Highest priority based on analysis done by Cleton et al.^45^

^c^ Only in countries of the Eastern Mediterranean Region.

A limited number of Zika virus serological tests have been described in the literature. Data from studies of Zika virus seroprevalence and diagnosis published from 1950 to 1980 show that complement fixation and haemagglutination inhibition tests show extensive cross-reaction between Zika and other flaviviruses.^65^^,^^66^ Despite some risk of cross-reactivity, the most specific serological method for flaviviruses are virus neutralization tests.^45^ More recent studies, described below, are of Zika virus serological tests developed non-commercially and based on ELISA using whole viral antigen or recombinant protein or on immunofluorescence assay. As these tests have had only limited validation, the laboratory community urgently needs better validation data for serology testing in the field.

#### Non-commercial tests

Antibody-capture ELISAs for IgM and IgG (with whole inactivated viral antigen produced on suckling mouse brains) were used to map antibody responses in 11 patients from the Yap Island outbreak.^43^ A similar technique using whole viral antigen produced in Vero cells, was used to describe the antibody response of a Guillain–Barré syndrome patient.^10^ In the case of a patient with primary Zika virus infection with no history of other flavivirus infection or vaccination, IgM antibody-capture ELISA was unexpectedly specific for Zika virus with no cross-reaction with other flaviviruses^43^ (I Leparc-Goffart, French National Reference Centre for Arbovirus, unpublished data, 2016). A case of Zika virus infection in Australia, imported from Cook Islands, was diagnosed by RT–PCR with a non-commercial Zika virus microsphere immunoassay for IgM and IgG, using recombinant Zika virus non-structural protein 1 (NS1). The patient showed seroconversion for Zika IgM and IgG between an acute-phase sample at day 2 after the onset of symptoms and a convalescent-phase sample at day 10.^15^ Zika virus cases imported to Europe were diagnosed using whole-virus immunofluorescence assay for Zika virus IgM and IgG to determine seroconversion or a fourfold titre increase between acute- and convalescent-phase serum samples.^19^^,^^21^^,^^22^ Virus neutralization tests have been reported in a few studies to confirm antibody responses detected by ELISA or immunofluorescence assay.^10^^,^^22^^,^^43^ In a patient with a primary flavivirus infection with Zika virus, comparative neutralization tests only showed neutralizing antibodies against Zika virus and not against the four dengue fever viruses. The interpretation of neutralization tests when a patient has already been infected by or vaccinated against another flavivirus is more complex. Even if the neutralization titre were higher for Zika virus than for other flaviviruses, only a few patients had a titre for Zika virus that was fourfold higher than for other heterologous flaviviruses.^43^

#### Commercial tests

To the authors’ knowledge three commercial tests are on the market at the time of writing this article or will be available soon. The Zika virus IgG and IgM detection kits of MyBioSource Inc. (San Diego, USA) use a double-antigen sandwich ELISA. No information on the type of antigens used or on the test specificity and sensitivity is given by the manufacturer.^67^ Biocan Diagnostics Inc. (Coquitlam, Canada) offers a rapid finger-prick assay based on a mix of the NS1 protein and envelope protein that can detect IgM and IgG antibodies. The company states a specificity of 99% but no specific details of the validation procedure are given.^68^ Euroimmun AG (Lübeck, Germany) offers both immunofluorescence assay and ELISA for IgM and IgG. The Euroimmun immunofluorescence assay is offered in a mosaic slide together with detection for Zika virus, chikungunya virus and four dengue virus serotypes. The information provided indicates cross-reactivity with antibodies directed against tick-borne encephalitis virus, West Nile virus and dengue viruses for both the IgG and IgM assays. Furthermore, validation data for the IgM and IgG Zika virus immunofluorescence assay indicate a wide range of specificities and sensitivities depending on the validation cohort. The given values are hard to interpret as the description of the cohorts is insufficient. The validation data should be interpreted with caution, as positivity was only rated at the cut-off dilution. This could mean that the specificity may be different (higher) than stated, as the results were not scored as end-titres. The use of end-titres would provide a window for differentiating the (cross) reactivity measured. The ELISA is based on recombinant NS1 protein which leads to a reduction of cross-reactivity with other flaviviral antibodies to maximal values of 18.8% (IgG) and 8.3% (IgM). Euroimmun is currently the only manufacturer providing detailed validation data that clearly address the above-mentioned difficulties with cross-reactivity in flavivirus serodiagnostics.^69^

### Biosafety

Zika virus is classified as a biosafety level 2 pathogen in the European Union (with the exception of the United Kingdom) and the USA. There are no inactivation data available that are specific for Zika virus. However, flaviviruses are typically inactivated by temperatures above 56 °C for at least 30 minutes, in solutions of pH ≤ 6, by ultraviolet light and by gamma-radiation and are known to be susceptible to disinfectants such as 1% sodium hypochlorite, 2% glutaraldehyde, 70% ethanol, 3–6% hydrogen peroxide and 3–8% formaldehyde.^70^^–^^74^

During the initial steps of molecular detection methods phenol guanidine isothiocyanate or chaotropic salts are added to the samples to extract the RNA. These reagents inactivate flaviviruses, and therefore the diagnostic procedure could continue in a laboratory with standard safety levels after the addition of these reagents.^75^

Although Zika virus is only a level 2 pathogen, laboratories should assess the additional risks for laboratory personnel who are pregnant, especially when the virus is cultured (e.g. in virus neutralization tests).

## Synthesis and conclusions

Box 1 summarizes areas for research and action that would address some of the knowledge gaps identified in this paper. We suggest that an international laboratory response is needed, which would include preparation of protocols for prospective studies to address the most pressing information needs. The knowledge obtained should be put into the public domain as soon as possible.

Box 1 **Areas for research and actions needed to address gaps in knowledge about Zika virus diagnostic testing**

• The following areas of research are suggested to address the knowledge gaps identified:

– Urgent assessment of the laboratory capacity and capability of countries to detect Zika virus.

– Conducting rapid and extensive field validation of the available molecular and serological tests in areas with Zika virus transmission and in areas unaffected by transmission but receiving returning travellers. Special focus should be on pregnant women.

– Monitoring the genomic diversity of circulating Zika virus strains to allow verification against operational molecular tests to ensure continuous sensitivity of testing.

– Conducting prospective studies into the Zika virus infection kinetics in the general population and in pregnant women. This should focus on determining the ideal types of diagnostic samples (e.g. whole blood, plasma, serum, urine, saliva, etc.), combinations and times of collection (stage of illness).

– Developing external quality assessments for both molecular and serological testing. Besides Zika-virus-positive samples, these panels should include: (i) a cross-reactive panel, consisting of viruses or antibodies to closely related viruses (dengue virus, yellow fever virus, West Nile virus) and (ii) a syndromic panel with pathogens or antibodies to diseases with a clinical manifestation similar to Zika virus (e.g. chikungunya virus, malaria, rickettsia). External quality assessments for molecular testing should address different types of diagnostic samples.

• The availability of reagents for diagnostic development (e.g. virus strains, virus antigens, quantified viral ribonucleic acid) needs to be facilitated. This could be achieved through initiatives such as the European Virus Archive^76^ and the future EVD-LabNet by the European Centre for Disease Prevention and Control.

• An international laboratory response is needed, which would include preparation of protocols for prospective studies to address the most pressing information needs.
